# Medical ethics in childbirth: a structural equation modeling approach in south of Iran

**DOI:** 10.1186/s12910-024-01072-6

**Published:** 2024-06-26

**Authors:** Moghaddameh Mirzaee, Firoozeh Mirzaee

**Affiliations:** 1 Institute for Futures Studies in Health, Kerman University of Medical Sciences, Kerman, Iran; 2 Reproductive and family health research center,, Kerman university of medical sciences, Kerman, Iran

**Keywords:** Medical ethics, Structural equation Model, Vaginal child birth, Attitude, Validation

## Abstract

**Background:**

The existence of a valid instrument to evaluate the attitude of mothers towards compliance with medical ethics during childbirth can lead to appropriate interventions to create a positive attitude. The purpose of this study is to determine the construct validity of the MEAVDQ (Medical Ethics Attitude in Vaginal Delivery Questionnaire).

**Methods:**

The study was carried out with 350 women. The main research instrument was MEAVDQ. This 59-item questionnaire comprises three parts A, B, J. Part A is concerned with the first principles. Part B deals with the second and third principles and part J addresses the fourth principle of medical ethics. Structural Equations Modeling (SEM) was used to determine the construct validity of MEAVDQ.

**Results:**

The results of SEM revealed that there was a positive correlation between structures A and B. The relationship between structures B and J was also positive and significant. On the other hand, there was a direct and indirect relationship between structures A and J. One-unit increase in structure A led to 0.16 (95% CI: 0.01, 0.33) direct increase in structure J. Also, one-unit increase score increases in structure A caused 0.39 indirect rise (95% CI: 0.26, 0.53) in structure J with the mediating role of the structure B.

**Conclusions:**

It can be suggested to midwifery policy maker and midwives that respect for the first principle of medical ethics and autonomy is the most important principle of medical ethics in childbirth. By respecting the autonomy of mothers, a positive birth experience can be created for them.

## Introduction

The considerations related to medical ethics in obstetric obligates midwives and obstetricians to psychologically and physically empower the pregnant woman. The applications of ethics in this area include clinical management of pregnancy, child birth, and newborn care. These principals include awareness of medically reasonable alternatives to pregnancy management, child birth and neonatal care, as well as their clinical benefits and risks [1]. Medical ethics are founded on the moral, religious, and philosophical concepts and principles of the society and are influenced by economics, policies, and law [2].

Obstetrical ethics stress women’s rights about autonomy and body integrity [3, 4]. According to the American Congress of Obstetricians and Gynecologists, pregnant women’s autonomous decisions should be respected and they are entitled to refuse medical interventions except under extraordinary circumstances [3]. The right of autonomy and bodily integrity are of paramount importance. The rights of a fetus as the future child are recognized as legitimate concerns and they grow in significance as the pregnancy progresses. The status of the embryo is vital for the ethical consideration of all techniques used in reproductive medicine [5]. Modern Protestant theology and bioethics defend the following attitude: the embryo is not an independent human being as is the newborn child, because it has to be accepted by the body of the mother and has to be nourished continuously by the mother connected to her bloodstream. The ethical right to be protected prenatally rises regularly with the age and therefore the development of the embryo [6]. The Nursing and Midwifery Council (NMC) Standards of Proficiency for Midwives command that understanding the meaning of informed consent and seeking informed consent are critical midwifery skills [7].

Regarding the concept of beneficence and nonmaleficence, in a study has showed, nearly all nurses and midwives stated that, they were trying to ensure the safety of patients, and prevent them from harm and possible dangers [8].

The concept of justice, as understood in ancient time and the era of Aristotle, calls for equal treatment of people [3]. Medical services should be provided to all women on a fair and efficient basis. However, the principles of harmless treatments and cares are also vital to the medicine [9].

The four principles approach could be applied to a diversity of situations as a workable template in cases such as childbirth [9, 10]. Respecting a patient’s autonomy is a key point to foster the decision-making capacities of autonomous individuals, which allows them to make rational and conscious choices. Healthcare providers are morally bound to act in such a way that promote the autonomy of their patients in cases like the childbirth process [9].

In most debates on this issue, solutions to ethical problems have been reduced to a simple invocation of the four principles without considering the inherent concept and meaning of these principles, as if a solution can be readily achieved just by appealing such principles. Clearly, it is desirable to interpret them in the context of a problem [9].

Obstetrical ethics within medical ethics are concerned with the rights of woman, fetuses and/or the future of children, their family, and obligations of the healthcare team [3, 11].

Many studies have investigated medical ethics in obstetrics. By adopting a global perspective, Mccullough et al. (2016) stressed the importance of health care justice and human rights in perinatal medicine. Chervenak et al. (2016) explored ethics and professional accountability, emphasizing the essential dimension of the planned home birth [1, 12–14]. They both clarified one of the medical ethics principles. Mccullough et al. (2016) expressed one model in medical ethics. This model highlights the identification and careful balancing of the perinatologist’s ethical obligations to pregnant, fetal, and neonatal patients. This model stands in sharp contrast to one-dimensional maternal-rights-based reductionist model of obstetric ethics, which is based only on the pregnant woman’s rights [13].

The existence of a valid questionnaire that can measure the attitude of mothers during childbirth towards the respect of medical ethics can lead to the application of suitable interventions to create a positive attitude and experience of mothers towards this issue. A positive experience of childbirth reduces the desire of mothers to choose a cesarean section in subsequent pregnancies. Our study aims to investigate the construct validity of MEAVDQ and the relationships between medical ethics principles in childbirth using structural equation modelling (SEM) and confirmatory factor analysis.

## Methods

### Study participants

This cross-sectional study was conducted from October 2020 to January 2021. 350 participants consisted of women referring to Kerman Hospital of Iran. According to MicyYoung study(2022), this sample size is sufficient for this study [15].

The inclusion criteria were as follows: [1] a low-risk pregnancy and childbirth [2], Have a vaginal delivery in the last 7 days. Moreover, a decision to withdraw from the study was seen as the exclusion criteria.

### Questionnaire

Medical ethics attitude in vaginal delivery questionnaire (MEAVDQ) was employed in this study. This 59-item questionnaire comprises three structures, A, B and J. Part A explains the first principle of medical ethics, (respect to Autonomy). It consists of 19- items. Part B discusses the second and third principles (respect to Beneficence, and non-maleficence). It consists of 27- items and part J explores the fourth principle of medical ethics (respect to Justice). It consists of 13- items. Structures A, B and J have 3, 7, 3 dimensions, respectively [16].

The dimensions of structure A include: Providing the necessary information, Mother’s privacy and Interaction with mother, Structure B include: The importance of the role of midwife, Ensuring the health of the fetus, Mother’s pain, Mother’s stress, Mother’s health, Mother’s need for pain reductions and Mother’s relaxation, Structure J include: Trust in the midwife, The necessity to meet the mother’s requests and the application of equal opportunities for every mother [16].

The desirable face and content validity of this questionnaire (CVR = 0.78, CVI = 0.89) have been confirmed. construct validity has been checked by exploratory factor analysis. construct validity, empirically, the conceptual boundaries are established by creating an expected pattern of convergent validity (showing that the scale correlates with other psychological measures to which it is conceptually similar) and discriminant validity (showing that the scale does not correlate with measures to which it is conceptually dissimilar) coefficients [17]. Three dimensions of part “A” explained 62.8% of variance in this part. Seven dimensions of part B justified 64% of variance in this part, and three dimensions of part J explained 51% of variance in this part. The reliability of this instrument has been demonstrated (ICC = 0.6–0.95) [16].

In this study we investigate the construct validity of MEAVDQ and relationships between medical ethics principles in childbirth using by structural equation modelling (SEM) and confirmatory factor analysis.

### Statistical analysis

The descriptive analysis consisted of frequency, percentage, mean and standard deviation. The multiple linear regression was used to explore the relationship between demographic variables and dimensions of vaginal delivery questionnaire.

SEM was also adopted to clarify the association between medical ethics principles and subcategories of these principles in the childbirth and construct validity. The causal relationship of variables is examined directly and indirectly in a SEM [18].

The coefficients in the SEM are interpreted as the Pearson correlation coefficient and the regression coefficients.

SPSS 20 and AMOS 18 were used for data statistical analysis. *p*-value below 0.05 was considered significant.

### Ethical consideration

This study was approved by the ethics committee of Kerman University of Medical Sciences (Reg. No. 96,000,327). After approval, a permit was issued to refer to the maternity ward of Hospital. The researcher explained the study objectives to the participants reassuring them about confidentiality of data and that they could leave the study at any time at will, Also, informed consent was obtained from participants.

## Results

The mean age of women was 26.87 ± 5.7. The majority of women (79%) had one or two pregnancies. Less than a third of women had a history of miscarriage, stillbirth, and preterm delivery (23.7%). As for the level of education, most women (73%) had only primary and middle school education. The other demographic information of participants in this study is shown in Table 1.

**Table 1:** Description of demographic information of participants

Qualitative variables		Frequency	Percentage
Job	Housewife	253	84.3
	Employee/Self employed	47	15.7
Educational level	Illiterate	43	14.3
	Under diploma	219	73
	College education	38	12.7
Gravidity	1	118	39.3
	2	119	39.7
	3	45	15
	4	12	4
	5	3	1
	6	2	0.7
	7	1	0.3
History of miscarriage, stillbirth and preterm delivery	Yes	229	76.3
	No	71	23.7
Spouse level of education	Illiterate	45	15
	Below diploma	217	72.3
	College education	38	12.7
Spouse job	Unemployed	27	9
	Employee	58	19.3
	Self-employment	215	71.7
**Quantitative variables**	**Mean ± S.D**	**Min**	**Max**
Age (year)	26.87 ± 5.7	16	42
Spouse age	31.12 ± 5.9	19	60

In structure A, the highest correlation was between two dimensions of “interaction with mother” and “provision of necessary information” (*r* = 0.58). In structure B, the greatest correlation was between two dimensions of “mother’s need for pain reduction” and “mother’s health” (*r* = 0.59). In structure J, the strongest correlation was between two dimensions of “trust in the midwife” and “the necessity of meeting the mother’s requests” (*r* = 0.35) (Table 2).

**Table 2:** Correlation between different dimensions of A, B, J in participants

Structure	Dimensions of structures		Correlation	*p*- value
A	Mother’s privacy	Providing the necessary information	0.49	< 0.0001
	Interaction with the mother	Providing the necessary information	0.58	< 0.0001
	Interaction with the mother	Mother’s privacy	0.52	< 0.0001
B	Ensuring the health of fetus	The importance of the role of midwife	0.50	< 0.0001
	Mother’s pain	The importance of the role of midwife	0.36	< 0.0001
	Mother’s stress	The importance of the role of midwife	0.57	< 0.0001
	Mother’s health	The importance of the role of midwife	0.39	< 0.0001
	Mother’s need for pain reduction	The importance of the role of midwife	0.41	< 0.0001
	Mother’s relaxation	The importance of the role of midwife	0.54	< 0.0001
	Mother’s pain	Ensuring the health of the fetus	0.38	< 0.0001
	Mother’s stress	Ensuring the health of the fetus	0.58	< 0.0001
	Mother’s health	Ensuring the health of the fetus	0.45	< 0.0001
	Mother’s need for pain reduction	Ensuring the health of the fetus	0.42	< 0.0001
	Mother’s relaxation	Ensuring the health of the fetus	0.50	< 0.0001
	Mother’s stress	Mother’s pain	0.29	< 0.0001
	Mother’s health	Mother’s pain	0.45	< 0.0001
	Mother’s need for pain reduction	Mother’s pain	0.48	< 0.0001
	Mother’s relaxation	Mother’s pain	0.38	< 0.0001
	Mother’s health	Mother’s stress	0.44	< 0.0001
	Mother need for pain reductions	Mother’s stress	0.45	< 0.0001
	Mother’s relaxation	Mother’s stress	0.51	< 0.0001
	Mother’s need for pain reduction	Mother’s health	0.59	< 0.0001
	Mother’s relaxation	Mother’s health	0.32	< 0.0001
	Mother’s relaxation	Mother’s need for pain reductions	0.41	< 0.0001
J	Trust in the midwife	The necessity to meet the mother requests	0.35	< 0.0001
	Equal opportunities for every mother	The necessity to meet the mother’s requests	0.04	0.50
	Trust in the midwife	Equal opportunities for every mother	0.05	0.40

As far as the correlation between demographic variables and structures is concerned, the only variable that was significantly related to structures A and B was the educational status of women. The mean score of A, women with college education was 8.29(95% CI: 2.52, 19.9) higher than illiterate women (*p* = 0.005). The mean score of B, women with under diploma was 3.63(95% CI: 0.04, 7.23) higher than illiterate woman (*p* = 0.04). Also, the mean score of B, women with college education was 7.53(95% CI: 2.32, 12.70) higher than illiterate woman (*p* = 0.005).

Structure J did not have any significant relationship with demographic variables (Table 3).

**Table 3:** The correlation between demographic variables and structures

Variables		A			B			J		
		Coefficient	95% Confidence Interval	*P*-value	Coefficient	95% Confidence Interval	*P*-value	Coefficient	95% Confidence Interval	*P*-value
Age		0.20	(-0.22, 0.63)	0.34	0.24	(-0.14, 0.62)	0.22	-0.24	(-0.58, 0.10)	0.16
Gravidity		0.44	(-1.12, 2.07)	0.60	0.83	(-0.65, 2.32)	0.27	1.26	(-0.07, 2.58)	0.06
Spouse age		-0.25	(-0.64, 0.14)	0.20	-0.24	(-0.59, 0.11)	0.19	0.07	(-0.24, 0.39)	0.66
Educational status	Illiterate	Ref	-	-	-	-	-	-	-	-
	Under diploma	2.36	(-1.58, 6.31)	0.24	3.63	(0.04, 7.23)	0.04	2.79	(-0.42, 5.90)	0.09
	College education	8.29	(2.52, 13.9)	0.005	7.53	(2.32, 12.7)	0.005	4.33	(-0.31, 8.98)	0.07
Job	Housewife	Ref	-	-	-	-	-	-	-	-
	Employee/ Self employed	-2.98	(-6.79, 0.18)	0.12	-3.2	(-6.58, 0.34)	0.08	2.03	(-1.06, 5.12)	0.19
History of miscarriage, stillbirthpreterm delivery	No	Ref	-	-	-	-	-	-	-	-
	Yes	2.14	(-0.87, 5.15)	0.16	0.89	(-1.86, 3.63)	0.53	1.05	(-1.39, 3.50)	0.39
Spouse level of education	Illiterate	Ref	-	-	-	-	-	-	-	-
	Under diploma	2.21	(-1.68, 6.13)	0.27	2.77	(-0.77, 6.33)	0.13	-2.03	(-5.21, 1.13)	0.20
	College education	-2.4	(-8.03, 3.23)	0.40	0.91	(-4.22, 6.04)	0.73	-1.64	(-6.22, 2.93)	0.48
Spouse job	Unemployed	Ref	-	-	-	-	-	-	-	-
	Employee	-3.65	(-8.91, 1.60)	0.17	-1.56	(-6.34, 3.22)	0.52	0.54	(-3.73, 4.81)	0.80
	Self-employed	-2.60	(-7.25, 2.05)	0.27	-2.62	(1.62, 1.47)	0.22	-1.30	(-5.08, 2.48)	0.50

To examine the correlation between structures, we used SEM and a self-designed model. These results are shown in Chart 1. The coefficients in the chart are the Pearson correlation coefficient and regression coefficients. In structure A, the highest correlation was found with the dimension of “interaction with the mother” (*r* = 0.84). In structure B, the strongest correlation was observed with the dimension of “Mother’s stress” (*r* = 0.78). In structure J, the greatest correlation was found with the dimension of “Trust in the midwife” (*r* = 0.92).

The results of SEM in Table 4 suggested that there was a direct relationship between structures A and B. With one unit increase in structure “A”, structure B increased directly by 0.77 unit (95% C.I: 0.72, 0.82).

**Table 4:** The results of the SEM derived from the corresponding model in Chart 1

Path	Type	Standardized Coefficient (95%C.I)	t-Statistics	*P*-value
A → B	Direct	0.77(0.72, 0.82)	31.3	< 0.0001
B → J	Direct	0.51(0.33, 0.67)	5.90	< 0.0001
A → J	Direct	0.16(0.01, 0.33)	2.01	0.04
A → J (A→B→J)	Indirect	0.39(0.26, 0.53)	5.72	< 0.0001
A → J	Total	0.55(0.48, 0.63)	14.61	< 0.0001

Also, the association between B and J structures was also direct and significant and structures A and J were directly and indirectly related. With one unit increase in structure “B”, structure J increased directly by 0.51 unit (95% CI: 0.33, 0.67). With one unit increase in structure “A”, structure J increased directly by 0.16 unit (95% CI: 0.01, 0.33). Also, one unit increase in structure A led to an indirect rise in structure J by 0.39 unit (95% CI: 0.26, 0.53) through the mediation of structure B. The indirect effect of structure “A” on J was greater than its direct effect (Table 4).

**Fig. 1:**
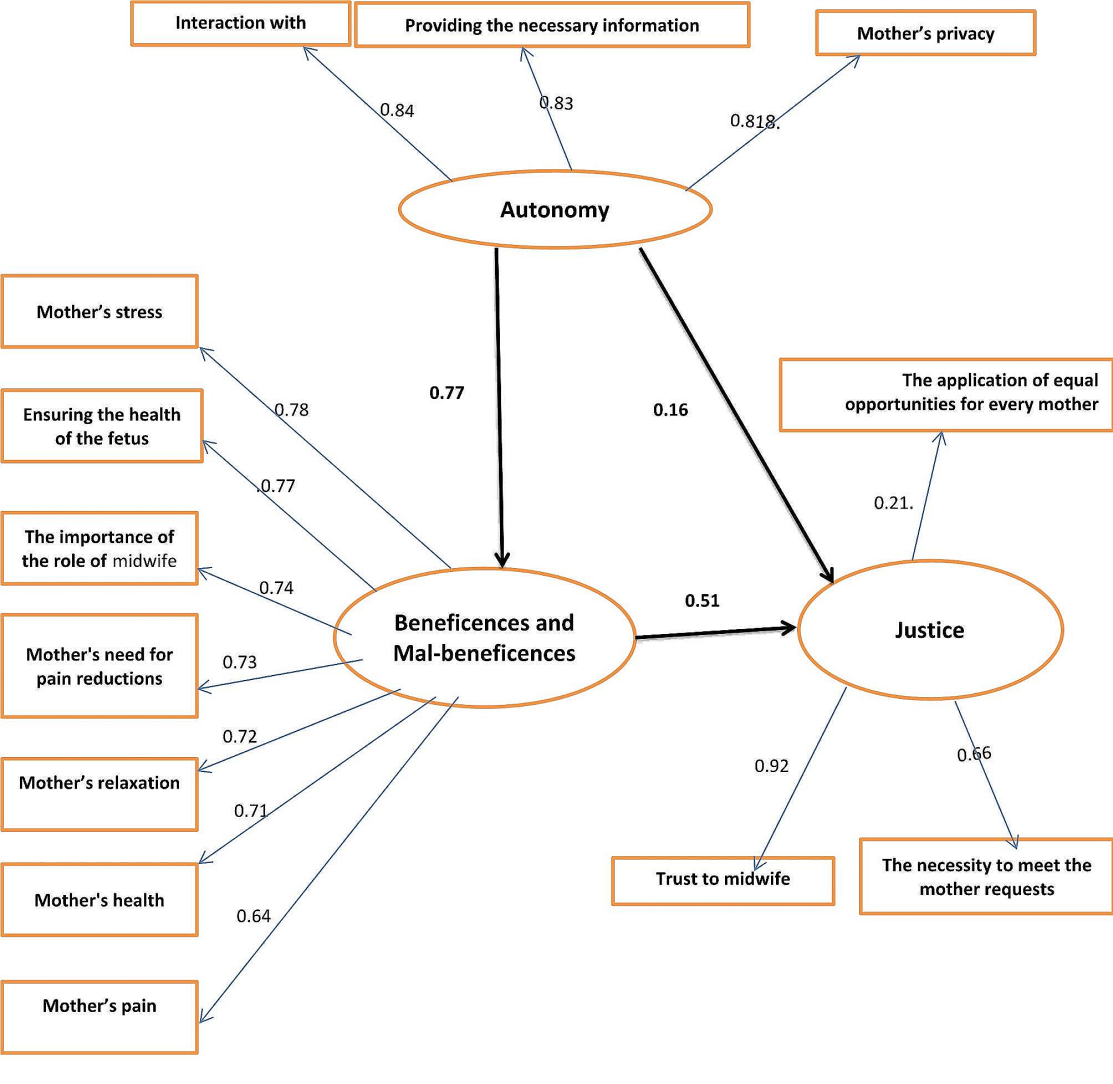
The structured model of relationship between Autonomy, Beneficences and Mal-beneficences and Justice in vaginal delivery subscales

Confirmatory factor analysis was performed to test the model Fig. 1. Chi-square was statistically significant (*p*-value < 0.0001), and the Chi-square/df ratio was 3.12 that is below than 5, indicating that the model fit the data well. GFI, AGFI and CFI indices were 0.95, 0.92 and 0.89 respectively that were close to or above than the recommended value of 0.90 and indicated good model fit. Also, the RMSEA of 0.04 indicated good model fit.

## Discussion

In the present study, we examined the construct validity of MEAVDQ through structural equation modelling (SEM) and confirmatory factor analysis. also, we proposed a new model that describes the relationship between medical ethics principles in childbirth. According to this model, respect to autonomy of medical ethics is the most important principle of medical ethics in childbirth, because by improving this principle, respect to Beneficence, and non-maleficence and respect to justice are enriched.

Some studies have shown that respecting patient autonomy is a core principle influencing physicians, patients, and family decision-making. When discussing advanced care plans or current decisions, physicians may start by identifying patients’ attitudes towards autonomy. Physicians who value the patient’s control over treatment choices tend to respect their preference. Elsewhere, the therapeutic approach will be imposed upon the patients. An initial approach would be to inquire whether the patients have completed their advance directive. It is recommended that physicians learn about patients’ motivations for completing a directive as a way of ascertaining their interests in autonomy and treatment preferences [19].

Patient age and race are two factors that sway their definition autonomy; older adults are more likely than younger patients to set priorities other than life prolongation, and to think in terms of care goals rather than specific treatment wishes [20]. In our study, however, women age did not influence their attitude towards medical ethics in childbirth. Most women in our study were young and of the same age. Racial and ethnic backgrounds also affect patients’ attitudes of autonomy [20, 21]. In this study, patients shared the same racial and ethnic backgrounds.

The educational status correlated with the first, second and third principles of medical ethics. Literacy plays a pivotal role in an individual’s attitude. A meta-analysis found that low health literacy was associated with poor health outcomes [22]. The results of pathway model suggested that providing valuable information to mothers and establishing a good relationship with them are essential components of “respect for the autonomy”. Disclosure of information relevant to the decision-making process promotes autonomy when care providers are ensure of their patients understanding [9].

Effective medical communication aims to enable physicians and their patients to foster an alliance regarding the adoption of the best therapeutic approach. In recent studies, a shared decision-making model has been promoted [23, 24], with the results exhibiting that sharing information about treatment options between physicians and patients helps reach consensus regarding the preferred option. The participation of patients in such decisions implies that they have control over their treatment decision making and can regain a sense of control and mastery over their disease or treatment. Doctors need to be attuned to patients’ desired level of participation in making decisions about treatment options [25].

This study reflected that attempt to reduce the mother’s labor pain fosters respect for second and third principles of medical ethics. Labor pain intensity is one of the most severe pains experienced by almost all women during vaginal labor, which may produce adverse effects on the mother and fetus [26, 27]. Pain relief based on mother’s request during labor ensures that the second and third principles of medical ethics are respected.

Physicians should be willing to understand and respect patients’ preferences and care goals [20]. Healthcare justice should be recognized as the basis for rights of humans to access healthcare and participate in decisions taken about one’s health [12, 28].

The results of SEM in path analysis revealed that the first principle was directly related to the second and third principles. Moreover, the second and third principles were directly and significantly correlated with the fourth principle. Further, there was a direct and indirect relationship between the first and fourth structures.

The principle of “respect for patient autonomy” is widely observed in modern medical ethics by physicians and other healthcare providers to foster the patient’s self- determination in medical care [3, 29].

### Limitations of the study

Data collected through a selfreport questionnaire may not accurately reflect the exact respondents’ views. Moreover, the study was conducted in a specific geographical area. Therefore, caution should be practiced in generalizing findings to other settings, populations, and areas.

## Conclusion

Based on the results of this study, this instrument has suitable the construct validity and it is recommended for interventional research on medical ethics in childbirth.

The results of this study suggested that respect for the first principle of medical ethics is the most important principle of medical ethics in childbirth, because by improving this principle, two other principles are enriched, so it can be said that effective interventions in improving the first principle of medical ethics in childbirth can be upgraded other principles of medical ethics in childbirth.

## Data Availability

The datasets used and/or analysed during the current study are available from the corresponding author on reasonable request.

## References

[1] Chervenak FA, McCullough LB (2016). Moral philosophy in perinatalology: a collaborative model for perinatal ethics. Semin Perinatol.

[2] Serour GI, Serour AG (2017). Ethical issues in infertility. Best Pract Res Clin Obstet Gynecol.

[3] Mercurio MR (2016). Pediatric obstetrical ethics: medical decision-making by, with, and for pregnant early adolescents. Semin Perinatol.

[4] Antiel RM (2016). Ethical challenges in the new world of maternal–fetal surgery. Semin Perinatol.

[5] Lanzone A (2013). Ethical issues in human reproduction: catholic perspectives. Gynecol Endocrinol.

[6] Birkhäuser M (2013). Ethical issues in human reproduction: protestant perspectives in the light of European Protestant and Reformed churches. Gynecol Endocrinol.

[7] Elf R, Nicholls J, Ni Y, Harris J, Lanceley A (2024). Consent practices in midwifery: a survey of UK midwives. Midwifery.

[8] Jafari H, Khatony A, Abdi A, Jafari F (2019). Nursing and midwifery students’ attitudes towards principles of medical ethics in Kermanshah, Iran. BMC Med Ethics.

[9] Lawson AD (2011). What is medical ethics?. Andrew D Lawson.

[10] One, Yun (2014). HW LJ. Perceptions about the Professional Ethics of EMT. Fire Sci Eng [Internet].

[11] Thomas R, Parker LS, Shiffman S (2021). The Ethics of Tobacco Harm reduction: an analysis of E-Cigarette availability from the perspectives of Utilitarianism, Bioethics, and Public Health Ethics. Nicotine Tob Research: Official J Soc Res Nicotine Tob.

[12] Chervenak FA, McCullough LB (2016). Healthcare Justice and human rights in perinatal medicine. Semin Perinatol.

[13] McCullough LB, Grünebaum A, Arabin B, Brent RL, Levene MI, Chervenak FA (2016). Ethics and professional responsibility: essential dimensions of planned home birth. Semin Perinatol.

[14] Chervenak FA, McCullough LB (2015). Ethics in perinatal medicine: a global perspective. Seminars Fetal Neonatal Med.

[15] Sim M, Kim SY, Suh Y (2022). Sample size requirements for simple and complex mediation models. Educ Psychol Meas.

[16] Mirzaee Rabor F, Taghipour A, Mirzaee M, Mirzaii Najmabadi K, Fazilat Pour M, Fattahi Masoum SH (2015). Developing a questionnaire for Iranian women’s attitude on Medical Ethics in Vaginal Childbirth. Nurs Midwifery Stud.

[17] Piedmont RL, Maggino F (2023). Construct validity. Encyclopedia of Quality of Life and Well-Being Research.

[18] Elizabeth C, Pinoa KD, Jackb B, Hendersonc D, Milanovicd S, Kalesane B (2018). Adolescent socioeconomic status and depressive symptoms in later life: evidence from structural equation models. J Affect Disord.

[19] Karasz A, Sacajiu G, Kogan M, Watkins L (2010). The rational choice model in family decision making at the end of life. J Clin Ethics.

[20] Winzelberg GS, Hanson LC, Tulsky JA (2005). Beyond autonomy: diversifying end-of-life decision-making approaches to serve patients and families. J Am Geriatr Soc.

[21] Frechman E, Dietrich MS, Walden RL, Maxwell CA (2020). Exploring the Uptake of Advance Care Planning in older adults: an integrative review. J Pain Symptom Manag.

[22] Rutherford EJ, Kelly J, Lehane EA, Livingstone V, Cotter B, Butt A (2018). Health literacy and the perception of risk in a breast cancer family history clinic. Surgeon: J Royal Colleges Surg Edinb Irel.

[23] Kasule OH (2013). Medical professionalism and professional organizations. J Taibah Univ Med Sci.

[24] Guraya SY, Guraya SS, Mahabbat NA, Fallatah KY, Al-Ahmadi BA, Alalawi HH (2016). The desired Concept maps and goal setting for assessing professionalism in Medicine. J Clin Diagn Research: JCDR.

[25] Stuij SM, Labrie NHM, van Dulmen S, Kersten MJ, Christoph N, Hulsman RL (2018). Developing a digital communication training tool on information-provision in oncology: uncovering learning needs and training preferences. BMC Med Educ.

[26] Dehcheshmeh FS, Rafiei H (2015). Complementary and alternative therapies to relieve labor pain: a comparative study between music therapy and Hoku point ice massage. Complement Ther Clin Pract.

[27] Hajiamini Z, Masoud SN, Ebadi A, Mahboubh A, Matin AA (2012). Comparing the effects of ice massage and acupressure on labor pain reduction. Complement Ther Clin Pract.

[28] Rigby FB. Ethics in the obstetric critical care setting. wiley blackwell; 2018.

[29] Collins SC, Chan E (2017). Sociocultural determinants of US women’s ethical views on various fertility treatments. Reprod Biomed Online.

